# A randomized controlled trial of a new intervention in early symptomatic syndromes eliciting neurodevelopmental clinical examinations: PR-ESSENCE

**DOI:** 10.1007/s00787-021-01837-z

**Published:** 2021-07-03

**Authors:** Mats Johnson, Carina Gillberg, Ingrid Vinsa, Gunnar Fransson, Lena Samuelsson, Klara Jakobsson, Sven Östlund, Elisabeth Fernell, Christopher Gillberg

**Affiliations:** Gillberg Neuropsychiatry Centre, Sahlgrenska Academy, University of Gothenburg, Kungsgatan 12A, 411 19 Gothenburg, Sweden

**Keywords:** PR-ESSENCE, Children, Adolescents, ADHD, Autism, ESSENCE

## Abstract

The need for effective intervention programs for youth with neurodevelopmental problems (ESSENCE) and challenging behaviour is great. This study examines Problem Resolution in ESSENCE (PR-ESSENCE), a newly developed model in which children and parents develop mutual problem resolution strategies. Ten-week randomized controlled trial of PR-ESSENCE for children and adolescents aged 5–18 years, compared to treatment as usual. Outcomes were assessed at baseline and randomized period endpoint. Primary outcome was the Clinical Global Impression—Improvement scale (CGI-I) rated by blinded assessors. Secondary outcomes were rated by parents—SNAP-IV, Eyberg Child Behavior Inventory (ECBI), Relationship Problems Questionnaire, Family Burden of Illness Module, and children—Beck Youth Inventories (BYI). ClinicalTrials.gov identifier: NCT03780413. The study enrolled 108 participants (active *n* = 72; controls *n* = 36, randomized 2:1), of whom 95 completed the randomized period. No clinically significant group differences were found in baseline characteristics. More than half had autism and 80% had ADD or ADHD. Large treatment effects were seen on CGI-I (ITT analysis, Effect Size 1.48). Treatment responders, much/very much improved on CGI-I, were 51.4% in active group and 5.6% of controls. Effect sizes were medium to large in parent ratings on SNAP-IV (ODD and ADHD symptoms), ECBI (behaviour problems), and in BYI child self-ratings of disruptive behaviour. PR-ESSENCE treatment improved global symptoms and functioning (CGI-I), behaviour problems, ADHD and ODD symptoms, and disruptive behaviour. Treatment effects were at least equivalent to those in previous studies of well-established Parent Management Training and Collaborative Problem Solving programs.

## Introduction

Autism and Attention-Deficit/Hyperactivity Disorder (ADHD) belong in a group of overlapping neurodevelopmental conditions now often referred to under the umbrella term of ESSENCE (Early Symptomatic Syndromes Eliciting Neurodevelopmental Clinical Examinations) [1]. They are still often diagnosed as separate disorders and treated with psychoeducation and behavioural interventions on the one hand and medication (including neuroleptics and stimulants) on the other. A prevailing, and difficult-to-treat, problem in both autism and ADHD—and in several of the other disorders in the group of ESSENCE (including Tourette syndrome and other tic disorders)—is the marked inability to control temper coupled with oppositional-defiant behaviours. Indeed, one study of young children [2] showed inability to control temper to be the single most common problem in ADHD. More than 95% of the whole group with ADHD had this “oppositional” symptom, which was even more prevalent than any of the features/symptoms considered diagnostic of ADHD “itself”. This emotional dysregulation, including deficits in emotional inhibition and flexibility, although not included in the diagnostic criteria for autism and ADHD, is reflective of executive function deficits, a cognitive area characterizing both these disorders [3–5]. Oppositional behaviours are often not well controlled by current interventions deemed appropriate for ADHD, autism and other ESSENCE.

The “Collaborative Problem Solving” model was developed by Greene [6] for the treatment of youth with oppositional behaviour, and is now referred to as Collaborative & Proactive Solutions (CPS). The aim of CPS is to help caregivers and children practice and learn problem solving strategies in collaboration. The theoretical basis of the model is that problematic behaviour is mainly caused by lagging skills, especially regarding frustration tolerance, flexibility and problem-solving ability. Cognitive, executive and theory of mind deficits make communication and mutual understanding problematic. The CPS model engages both the child and the parents in training and may therefore have potential to give long-term improvement of skills in handling problematic situations and to change the mindset towards mutual problem-solving strategies.

The CPS model was evaluated in a US trial [7] comparing CPS to Barkley’s [3] Parent Management Training program (PMT) in 47 children aged 4–12 years with Oppositional Defiant Disorder (ODD) and affective dysregulation. Outcomes were similar between the two methods in most measures post-treatment and at 4 months follow-up. A recent larger randomized controlled US trial [8] enrolled 134 children with ODD, aged 7–14 years, who were randomized to three groups (CPS, PMT or Waitlist Control). Many children also had comorbid ADHD and/or Anxiety Disorder, but children with Autism Spectrum Disorder (ASD) were excluded. Both treatment groups had equivalent results in treatment response and remission of diagnostic status (46.7% in the CPS group were much to very much improved on the Clinical Global Impression-Improvement Scale (CGI-I), i.e., reached a CGI-I score of 1–2), and both treatments were superior to the Waitlist Control condition.

The first Swedish study of CPS [9], a small open pilot trial of three months CPS for 17 families and children aged 7–13 years, with ADHD, ODD and challenging explosive behaviour, showed promising results. At post-treatment 53% of the children were much or very much improved on global symptom and function ratings (CGI-I score 1–2), and after another six months when the children with severe residual ADHD symptoms also had received stimulant medication, 81% of all reached CGI-I levels of 1–2.

Thus, previous research has established CPS as an effective treatment for ODD in youth, but effectiveness in children with various neurodevelopmental disorders has not yet been documented. Challenging behaviours (oppositional, explosive and disruptive) constitute a major problem among youth with neurodevelopmental disorders/ESSENCE and the need for treatment methods is great.

In our own open study [9], the therapists delivering the intervention were highly skilled in the field of autism, ADHD and other ESSENCE. In clinical work, after completion of that study, our group reached the conclusion that, in order to be useful for families with severely impairing ESSENCE (not just mild-moderate ADHD with oppositional behaviours), the CPS model would have to be considerably modified and delivered in a more flexible way by experienced clinicians with “hands-on-expertise” in the field of ESSENCE. After a number of research meetings and seminars, we designed a new model that we now refer to as PR-ESSENCE (Problem Resolution in ESSENCE). We wanted to test this model in a randomized controlled study, the results of which are presented and analyzed below. The trial has a broad focus on complex neurodevelopmental disorders with a view to more closely reflecting the patient populations encountered in everyday clinical practice, in contrast to the narrow diagnosis-restricted focus in many existing clinical trials. During the early phases of this study, Dr. Greene gave advice and supervision to our treatment team, and the whole PR-ESSENCE model would not have been developed without this input.

### Objectives

This study aimed at investigating if the PR-ESSENCE model could (1) reduce challenging behaviours—outcome measures were the rating scales CGI-I, CGI-S, SNAP-IV, ECBI, FBIM, RPQ—see below, (2) improve the children’s emotional wellbeing and self-concept—outcome measure was the Beck Youth Inventories, and (3) help the child and parents to solve specific problem situations—outcome measure was a Problem Rating Scale developed by us for this study.

Results relating to objectives 1 and 2 will be presented in this paper. Results related to objective 3 will be reported in a future publication.

## Methods

As discussed in the Introduction, extensive clinical experience of treatment of children with complex neurodevelopmental disorders (mainly autism and/or ADHD) has led the therapists in our team (two special education teachers, a psychologist and a study nurse) to make major ESSENCE-adapted modifications of CPS, particularly regarding verbal and visual communication, clarified structure in time, environment and transitions, predictability in plans and activities, and focusing on the child’s interests to facilitate interaction. It was essential to adapt the intervention to children with severe flexibility problems and difficulties to take other people’s perspective, and to the age and level of understanding of each child. The intervention that we have tested, therefore, is not CPS, but PR-ESSENCE. During the treatment period the parents and children met the therapists at approximately 10 (range 8–12) 1-h weekly visits. The PR-ESSENCE treatment aims to help the child and the parents to change their mindset and actions. During the treatment efforts were put on building a positive relation to the family to improve motivation and provide a basis with hope for good outcomes. Of prime importance was that the child felt listened to, creating good conditions for them to describe what they perceived as problematic. Visual aids such as drawing while talking were used for clarification, helping the child to understand situations and consequences, and to see benefits of change. The child and parents were guided to understand the specific mechanisms behind problem situations and to suggest solution strategies. They practiced the problem-solving strategies at home between sessions, focusing on one problem at a time, and documented their daily progress in a diary. The results were evaluated at each visit, and solutions were adjusted if needed. The parents were educated about the child’s strengths and difficulties, helping them to adapt expectations and communication, and to focus on predictability and structure.

Our study was designed to investigate the effects of PR-ESSENCE for challenging behaviours in a well-defined clinical sample of children with complex neurodevelopmental disorders. Participants were children and adolescents aged 5–18 years, with any neurodevelopmental diagnosis, and with intellectual level in the range above the intellectual disability level according to WISC or WPPSI-test [10–12] and clinical judgment, since children with intellectual disability might need more adaptations of the method to understand and use it.

Participants taking psychoactive medication could be included if the medication had been unchanged for at least one month before baseline and remained unchanged during the treatment period. Exclusion criteria were bipolar disorder, psychosis, or other unstable psychiatric or medical disorders, or substance use.

**Fig. 1:**
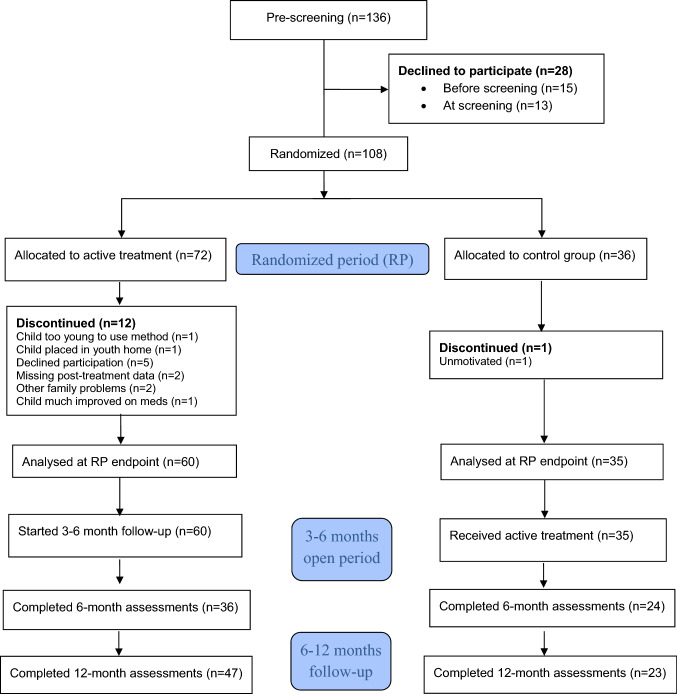
Flow of participants

After comprehensive neurodevelopmental/neuropsychiatric assessment and diagnosis according to DSM-5 criteria, performed by experienced teams at our centre or at the Child and Adolescent Psychiatry centres in Gothenburg, 136 children and adolescents with complex neurodevelopmental disorders/ESSENCE (i.e., ASD, ADHD, ASD + ADHD, learning disorders, dyslexia, etc.) and challenging behaviours were invited to participate in the trial between March 1, 2014 and Dec 8, 2017. Of these, 28 families declined participation before or at screening. The remaining 108 participants were enrolled and randomized 2:1 (computer-generated unstratified block randomization by an independent researcher) to a PR-ESSENCE treatment group and a control group, in a parallel group design (Fig. 1). The treatment group received PR-ESSENCE for 10 weeks. The control group received” Treatment As Usual (TAU)” (i.e., psychoeducation to parents and children and information to the school about individual needs for support and adaptations, which is given to all families after neurodevelopmental/neuropsychiatric assessment at our centre, and if indicated also psychoactive medication, which was kept stable during the trial, see inclusion criteria above). After the randomized period the control group received ten weeks of PR-ESSENCE. Outcome measures were collected at baseline, at the end of the randomized period, and after 6 months and 1 year. Follow-up endpoint was Jan 15, 2019.

### Outcome measures

To obtain results from different perspectives, ratings from four sources were used, i.e., rating by blinded investigators (two physicians with extensive experience in neuropsychiatric clinical research and CGI rating), parents, children and therapists. In this paper we report mainly the results from the randomized period. The long-term outcomes will be presented in detail in forthcoming publications. CGI-I is the primary outcome measure. CGI-S, parent ratings, child self-ratings and therapist ratings are secondary outcome measures. All the following measures were collected at baseline (except the CGI-I which measures post-baseline improvements), after the randomized 10-week treatment/TAU period (10 weeks), and after 6 months and 1 year.

### Ratings by blinded investigator (CGI-I, CGI-S)

The Clinical Global Impression (CGI) scales were developed by Guy et al. in 1976 [13] as general global ratings for medical conditions and have been used in a great number of clinical trials, including many treatment studies in ADHD [14]. In our trial, rating was based on interviews with parents and child considering challenging behaviours and functional impairment in several environments (school, with peers, family). CGI-I rates the global improvement or worsening of the patient’s condition compared to baseline, and thus gives an indication of how clinically meaningful an improvement is. The scale is scored from 1—very much improved; 2—much improved; 3—minimally improved; 4—no change; 5—minimally worse; 6—much worse, to 7—very much worse. Clinical response is defined by CGI-I ratings of 1–2. CGI-S rates the global symptom severity (scale from 1 to 7; 1 not at all ill, 2 borderline, 3 mildly ill, 4 moderately ill, 5 markedly ill, 6 severely ill, and 7 extremely ill).

### Parent ratings

The short version of SNAP-IV [15, 16] used in our study includes the DSM-IV ADHD (18 items) and ODD (8 items) criteria rated 0 (not at all), 1 (just a little), 2 (quite a bit) or 3 (very much), yielding an ADHD maximum score of 54 and an ODD maximum score of 24. The parent-rated Eyberg Child Behavior Inventory (ECBI) is designed to measure the frequency and severity of disruptive behaviours in home and school in children 2–16 years old, and how much of a problem the parents find the behaviour to be, with a 7-point Intensity Scale and a Yes/No Problem Scale [17]. The scale contains 36 items. A score of 131 or higher on the Intensity Scale suggests a significant problem. A score of 15 or higher on the Problem Scale indicates that the parent is significantly distressed by the behaviour. The ECBI has been shown to be a sensitive measure of treatment effects on disruptive behaviour problems [18]. The Family Burden of Illness Module (FBIM) is a 6-item measure of family stress and burden [19]. It was developed by Riley et al. [20], then called Family Stress Index (FSI). Items are scored from 0 (never), 1 (almost never), 2 (sometimes), 3 (almost always), to 4 (always), with a maximum score of 24. The Relationship Problems Questionnaire (RPQ), developed by Minnis et al. [21], probes both inhibited and disinhibited characteristics of attachment disorders. Rating is scored based on how similar the child´s behaviour is to the item statements, from 0 (not at all like), 1 (a bit like), 2 (like), and to 3 (exactly like).

### Child self-ratings

The children were interviewed with the Beck Youth Inventories (BYI), which includes symptom ratings of depression, anxiety, irritability, disruptive behaviour, and self-concept for children and adolescents 7–18 years old [22].

### Therapist ratings

A Problem Rating Scale (PRS) was developed for this study to describe the various types of problem situations that occurred, and the proportion of these which were partially or completely solved during PR-ESSENCE treatment. These results will be reported in a future publication.

### Statistical analysis

Sample size calculation indicated that with power 0.90 and significance level of 0.05, a sample of 102 participants would be required to detect a medium effect size (Cohen’s *d* 0.6), which would represent a reduction in symptom loads large enough to be readily noticeable in everyday settings and may therefore be considered as a clinically meaningful effect. All efficacy analyses were performed between the PR-ESSENCE group and the control group, unadjusted for confounders. The primary efficacy analysis was performed on all randomized subjects (Intention-to-Treat (ITT) population) with baseline values carried forward to endpoint for dropouts, and on the Full Analysis Set (FA set) defined as all randomized subjects with any baseline and any end of treatment measurements. Secondary analyses were performed on the FA set without any imputation of missing data, and sensitivity analyses with Stochastic Imputation. Since normally distributed data could not be expected in the sample non-parametric tests were used for all statistical analyses. For comparison between the groups Fisher’s non-parametric permutation test was used for continuous and interval scales variables, Mantel–Haenszel Chi-square test for ordered categorical variables, Fisher’s exact test for dichotomous variables and Pearson chi-square for non-ordered categorical variables. For all continuous and interval scaled variables the mean difference between the two groups with 95% CI is given based on Fisher’s non-parametric permutation test. For dichotomous variables the risk difference with 95% CI and the risk ratio with 95% CI are presented. Effect sizes (ES) were calculated as the difference in mean scores between PR-ESSENCE group and controls, divided by the pooled standard deviation. The distribution of continuous and interval scaled variables is reported as mean, SD, median, minimum and maximum and distribution of categorical variables as numbers and percentages.

## Results

Of the 136 families invited to participate in the study, 28 families declined participation before (*n* = 15) or at screening (*n* = 13), due to family reasons, lack of time, or moving to another area (Fig. 1). After screening the remaining 108 families were enrolled and randomized 2:1 to PR-ESSENCE treatment or to control group (active *n* = 72, control *n* = 36). The FA set was defined as all participants who completed baseline and at least one post-baseline assessment, i.e., all families who completed the post-treatment/control period assessment after 10 weeks (*n* = 95, active 60, controls 35). No significant differences were found in demographics and baseline characteristics of this population (Table 1), with the exception of Developmental Coordination Disorder (DCD), found in four children in the control group and none in the active group, but this difference did not correlate with the primary outcome. Also Generalized Anxiety Disorder (GAD) was present in four children in the control group, but none in the active group. These patients had somewhat worse CGI-I outcome than those without GAD, but due to the small number of patients an adjusted analysis was not expected to change the primary outcome significantly. Mean age was 10.5 years (active) and 10.4 years (controls). Most children had full school attendance, but 18.3% (active) and 14.3% (controls) had partial or no school attendance. A large majority had problems in school, at home, and social interaction problems with peers (Table 1). The most frequent neurodevelopmental diagnoses were ADHD Inattentive Presentation (“ADD”) or Combined Presentation (active and controls 80%) and ASD (active 53.3%, controls 60%), and many of these had a combination of ADD/ADHD and ASD (active and controls 40%). Although many of the children and adolescents in the study scored high on oppositional defiant symptoms and behaviours, we considered that in most cases these were best “explained” by the basic impairments associated with their main diagnoses of autism and ADHD and have therefore not assigned separate diagnoses of ODD in such cases.

**Table 1:** Demographics and baseline characteristics. PR-ESSENCE vs Controls (FA set)

	Variable	Active (*n* = 60)	Control (*n* = 35)	*p* value
Gender	Male	19 (31.7%)	15 (42.9%)	
	Female	41 (68.3%)	20 (57.1%)	0.38
Age		10.5 (2.8)	10.4 (3.0)	0.62
		10 (6; 18)	10 (6; 17)	
Child living with	Two parents living together	39 (65.0%)	26 (74.3%)	
	Two parents living separately	17 (28.3%)	5 (14.3%)	
	Single parent	2 (3.3%)	4 (11.4%)	
	Fosterhome	2 (3.3%)	0 (0.0%)	0.13
Number of siblings	1	38 (63.3%)	26 (76.5%)	
	2	21 (35.0%)	8 (23.5%)	
	3	1 (1.7%)	0 (0.0%)	0.16
Education	Full attendance	49 (81.7%)	30 (85.7%)	
	Partial attendance	9 (15.0%)	4 (11.4%)	
	No attendance	2 (3.3%)	1 (2.9%)	0.65
Problems in school	No problems	4 (6.7%)	3 (8.6%)	
	Some problems	22 (36.7%)	16 (45.7%)	
	A lot of problems	31 (51.7%)	16 (45.7%)	0.22
Problems at home	No problems	0 (0.0%)	1 (2.9%)	
	Some problems	7 (11.9%)	2 (5.7%)	
	A lot of problems	39 (66.1%)	30 (85.7%)	0.17
Social interaction problems with peers	No problems	12 (20.7%)	7 (20.0%)	
	Some problems but has friends	44 (75.9%)	24 (68.6%)	
	Marked problems	2 (3.4%)	4 (11.4%)	0.42
Neuropsychiatric diagnoses	ADD-ADHD	48 (80.0%)	28 (80.0%)	1.00
	ASD	32 (53.3%)	21 (60.0%)	0.68
	ODD	7 (11.7%)	2 (5.7%)	0.57
	OCD	4 (6.7%)	1 (2.9%)	0.78
	Depression	2 (3.3%)	0 (0.0%)	0.79
	Anxiety, unspecified	9 (15.0%)	2 (5.7%)	0.30
	Learning difficulties	6 (10.0%)	2 (5.7%)	0.75
	DCD	0 (0.0%)	4 (11.4%)	0.033
	Tourette	5 (8.3%)	3 (8.6%)	1.00
	Dyslexia	2 (3.3%)	6 (17.1%)	0.054
	Bipolar disorder	0 (0.0%)	0 (0.0%)	1.00
	Generalized anxiety (GAD)	0 (0.0%)	4 (11.4%)	0.033
	Other	12 (20.0%)	7 (20.0%)	1.00
Family history (1st and 2nd degree relatives)	ADHD	15 (25.0%)	5 (14.3%)	0.33
	ASD	11 (18.3%)	7 (20.0%)	1.00
	ODD	0 (0.0%)	2 (5.7%)	0.27
	OCD	0 (0.0%)	1 (2.9%)	0.74
	Depression	12 (20.0%)	4 (11.4%)	0.43
	Anxiety	4 (6.7%)	0 (0.0%)	0.31
	Substance Use	2 (3.3%)	1 (2.9%)	1.00
	Bipolar disorder	2 (3.3%)	1 (2.9%)	1.00
	Torurette	2 (3.3%)	1 (2.9%)	1.00
	DCD	4 (6.7%)	3 (8.6%)	1.00
	Dyslexia	0 (0.0%)	1 (2.9%)	0.74
	Eating disorder unspec	0 (0.0%)	0 (0.0%)	1.00
Medication at baseline	No medication	26 (52.0%)	15 (44.1%)	
	Methylphenidate	7 (12.0%)	13 (37.0%)	
	Long-acting amphetamine	9 (18.0%)	5 (14.7%)	
	Atomoxetine	3 (6.0%)	0 (0.0%)	
	Guanfacine	2 (4.0%)	0 (0.0%)	
	Mood stabilizers	1 (2.0%)	1 (2.9%)	
	SSRI	2 (4.0%)	0 (0.0%)	

*ADD* ADHD inattentive presentation, *ADHD* attention deficit hyperactivity disorder, *ASD* autism spectrum disorder, *ODD* oppositional defiant disorder, *OCD* obsessive compulsive disorder, *DCD* developmental coordination disorder, *SSRI* selective serotonin reuptake inhibitor

For categorical variables *n* (%) is presented. For continuous variables Mean (SD)/Median (Min; Max)/*n* = is presented for comparison between groups Fisher’s Exact test (lowest 1-sided *p*-value multiplied by 2) was used for dichotomous variables, Mantel–Haenszel Chi Square test for ordered categorical variables, Chi Square test for non-ordered categorical variables and Mann–Whitney *U*-test for continuous variables

During the 10-week randomized period, 12/72 families (16.7%) in the active group discontinued the study (Fig. 1) for the following reasons; child too young to use the method (*n* = 1), child placed in youth treatment home due to severe problems (*n* = 1), child declined participation (*n* = 5), family did not complete post-treatment assessment (*n* = 2), other family problems (*n* = 2), child so much improved on medication that family saw no need of PR-ESSENCE treatment (*n* = 1). In the control group one family (2.8%) discontinued due to sufficient improvement on medication.

A total of 60 families (55.6%; 36 active, 24 controls) completed assessments at 6 months, and 70 families (64.8%; 47 active, 23 controls) completed the 1-year follow-up (Fig. 1).

In the FA set 44% (42/95) of the children [40% (24/60) in the active group and 45% (19/35) of controls] received stable medication before and during the randomized period in accordance with the inclusion criteria. The medication was mainly stimulants (active group methylphenidate *n* = 7, amphetamine *n* = 9; control group methylphenidate *n* = 13, amphetamine *n* = 5 (Table 1). A few patients in the active group received atomoxetine or guanfacine or SSRI (sertraline), and one patient each in the active and control groups took mood stabilizers (risperidone, valproate). The remaining participants had no medication.

### Primary efficacy analysis

At the randomized period endpoint (10 weeks), ITT analysis of all randomized subjects demonstrated that the PR-ESSENCE treatment group was significantly more improved than controls on the blinded investigator-rated CGI-I scores (mean active 2.61, controls 3.83, mean difference − 1.22; 95% CI − 1.56; − 0.88, ES 1.48, *p* < 0.0001). FA-set analysis showed a similar effect size (mean active 2.53, controls 3.86, mean difference − 1.32; 95% CI − 1.68, − 0.95; ES 1.53, *p* < 0.0001). Treatment responders, i.e., those much or very much improved on the investigator-rated CGI-I with only mild symptoms remaining (CGI-I 1–2), were 51.4% (37/72) in the active group and 5.6% (2/36) in the control group (mean difference 45.8; 95% CI 30.0, 61.7, *p* < 0.0001) (Table 2).

**Table 2:** Primary efficacy analysis

Follow-up	Variable	Active (*n* = 72)	Control (*n* = 36)	p-value	Difference between groups Mean (95% CI)	Effect size
10 weeks	CGI-I global improvement	2.61 (0.83) 2 (1; 6) *n* = 72	3.83 (0.81) 4 (2; 5) *n* = 36	< 0.0001	− 1.22 (− 1.56; − 0.88)	1.48
	Non-responders (CGI-I 3–4)	35 (48.6%)	34 (94.4%)		− 45.8 (− 61.7; − 30.0)	
	Responders (CGI-I 1–2)	37 (51.4%)	2 (5.6%)	< 0.0001	45.8 (30.0; 61.7)	

Change from baseline to 10 weeks. PR-ESSENCE vs Controls (ITT population). Baseline values were carried forward to endpoint for dropouts

For categorical variables *n* (%) is presented. For continuous variables Mean (SD)/Median (Min; Max)/*n* = is presented for comparison between groups Fisher’s Exact test (lowest 1-sided *p*-value multiplied by 2) was used for dichotomous variables, and Fisher’s Non-Parametric Permutation Test was used for continuous variables. The confidence interval for dichotomous variables is the unconditional exact confidence limits. If no exact limits can be computed the asymptotic Wald confidence limits with continuity correction are calculated instead. The confidence interval for the mean difference between groups is based on Fishers non-parametric permutation test. Effect Size is Difference in Mean/Pooled SD

After the randomized period the control group received PR-ESSENCE treatment, which means that the rest of the study was open-label. At the six-month follow-up 62% (37/60) of all who completed the period were treatment responders, and after 1 year 63% (44/70). The 6- and 12-month results will be reported in more detail in a future publication.

### Secondary efficacy analysis

*Baseline* At baseline the active and control groups had similar scores on all secondary variables with the exception of the ECBI intensity score, which was significantly higher in the active group (FA-set analysis). This difference did not correlate with the primary outcome variable and is, therefore, not considered as a confounder (Table 3).

**Table 3:** Baseline scores of secondary efficacy variables

Baseline	Variable	Active (*n* = 60)	Control (*n* = 35)	*p*-value	Difference between groups Mean (95% CI)
CGI-S	CGI severity of illness	4.68 (0.75) 5 (3; 7) *n* = 60	4.60 (0.95) 5 (2; 6) *n* = 35	0.73	0.083 (− 0.273; 0.429)
BYI	Beck Youth Inventories				
	Anxiety	66.9 (26.5) 70.5 (3; 99.9) *n* = 60	64.9 (30.3) 74.1 (12; 99.6) *n* = 35	0.75	1.94 (− 9.98; 13.56)
	Depression	72.1 (26.2) 79.6 (1.9; 99.8) *n* = 60	63.4 (31.8) 74.6 (1.9; 99.7) *n* = 35	0.16	8.77 (− 3.54; 20.69)
	Anger	79.4 (25.5) 89.9 (4.2; 99.8) *n* = 60	71.6 (29.5) 84.3 (7.9; 99.4) *n* = 35	0.18	7.76 (− 4.07; 18.94)
	Disruptive behaviour	76.0 (24.2) 82.1 (13.1; 99.1) *n* = 60	69.9 (27.6) 78.2 (13.1; 98.4) *n* = 35	0.26	6.14 (− 4.69; 16.83)
	Self-concept	36.3 (27.6) 29.5 (1; 97.6) *n* = 60	40.2 (31.0) 26.8 (1.4; 93.9) *n* = 35	0.53	− 3.88 (− 16.05; 8.51)
SNAP-IV	ADHD	33.7 (10.2) 34.5 (2; 53) *n* = 60	30.2 (11.2) 31 (1; 48) *n* = 35	0.12	3.56 (− 0.90; 8.00)
	ODD	16.3 (6.1) 16.5 (0; 28) *n* = 60	14.4 (6.7) 15 (3; 24) *n* = 35	0.15	1.95 (− 0.75; 4.55)
ECBI	ECBI intensity scale	149.5 (32.9) 146.5 (65; 209) *n* = 60	136.1 (28.1) 136 (66; 197) *n* = 35	0.049	13.5 (0.1; 26.6)
	ECBI Problem scale	19.2 (6.5) 19 (3; 31) *n* = 60	17.0 (6.5) 16 (5; 33) *n* = 35	0.12	2.22 (− 0.53; 4.95)
FBIM	Family burden of illness	14.1 (5.2) 14 (3; 24) *n* = 60	15.6 (5.5) 16 (4; 24) *n* = 35	0.19	− 1.50 (− 3.72; 0.71)

PR-ESSENCE vs Controls (FA set). Stochastic Imputation

For continuous variables Mean (SD)/Median (Min; Max) / *n* = is presented for comparison between groups the Fisher´s Non Parametric Permutation Test was used for continuous variables. The confidence interval for the mean difference between groups is based on Fishers non-parametric permutation test

*Investigator ratings* Mean baseline scores on CGI-S were in the moderately to markedly ill range (active 4.68, controls 4.60).

*Child self-ratings* BYI scores at baseline were in the moderately to extremely elevated range on depression, anxiety, anger and disruptive behaviour, and below average on self-concept.

*Parent ratings* The baseline SNAP-IV ADHD and ODD scores were moderately to markedly elevated. The ECBI intensity and problem scale scores were in the range of problems causing significant distress. The FBIM scores indicated considerable family distress.

### Change from baseline to 10 weeks

Analyses were made on the FA set without imputation of missing data (Table 4).

**Table 4:** Secondary efficacy analysis

Variable	Active (*n* = 60)	Control (*n* = 35)	*p*value	Difference between groups Mean (95% CI)	Effect size
Change of CGI severity of illness (CGI-S)					
Improvement	38 (63.3%)	5 (14.3%)			
Unchanged condition	20 (33.3%)	26 (74.3%)			
Deterioration	2 (3.3%)	4 (11.4%)	**< 0.0001**		
Beck Youth Inventories					
Anxiety	− 1.06 (25.55) 0 (− 59.1; 59.2) (− 8.72; 6.50) *n* = 45	0.348 (28.678) − 1.9 (− 78.9; 87.9) (− 11.350; 12.133) *n* = 25	0.85	− 1.41 (− 14.52; 12.03)	0.053
Depression	− 4.01 (23.19) − 1.7 (− 58.9; 42.6) (− 10.90; 3.00) *n* = 45	2.12 (28.11) 0 (− 61.5; 98) (− 9.00; 13.69) *n* = 25	0.34	− 6.13 (− 18.48; 6.47)	0.245
Anger	− 1.78 (22.02) − 0.2 (− 70.2; 53.6) (− 8.40; 4.99) *n* = 44	0.712 (23.095) − 3.2 (− 32.2; 92) (− 7.800; 9.900) *n* = 25	0.66	− 2.49 (− 13.55; 8.89)	0.111
Disruptive behaviour	− 8.56 (19.18) − 5.3 (− 57; 48.6) (− 14.40; − 2.67) *n* = 44	0.624 (15.909) 0 (− 36.1; 33.8) (− 5.992; 7.164) *n* = 25	**0.044**	− 9.18 (− 18.31; − 0.24)	**0.508**
Self-concept	6·71 (20.73) 3·2 (− 37.1; 47.3) (0.38; 12.99) *n* = 44	− 0.337 (26.629) − 3.5 (− 58.3; 57.7) (− 11.540; 11.037) *n* = 24	0.23	7.04 (− 4.50; 18.82)	0.307
SNAP-IV					
ADHD	− 5.78 (7.51) − 5 (− 29; 7) (− 7.72; − 3.85) *n* = 60	− 1·1 (6.43) − 0.5 (− 13; 10) (− 3.64; 0.83) *n* = 34	**0.0057**	− 4.37 (− 7.40; − 1.33)	**0.612**
Attention deficit	− 3.30 (5.00) − 3 (− 21; 5) (− 4.59; − 2.00) *n* = 60	− 0.559 (3.917) 0 (− 9; 8) (− 1.929; 0.833) *n* = 34	**0.0058**	− 2.74 (− 4.73; − 0.77)	**0.591**
Hyperactivity	− 2.53 (4.70) − 1 (− 13; 9) (− 3.76; − 1.31) *n* = 60	− 0.882 (4.382) − 0.5 (− 11; 10) (− 2.389; 0.667) *n* = 34	0.100	− 1.65 (− 3.62; 0.30)	0.360
Oppositional defiance	− 3.97 (5.07) − 4 (− 17; 8) (− 5.27; − 2.66) *n* = 60	− 1.24 (5.02) − 1 (− 13; 11) (− 3.00; 0.50) *n* = 34	**0.016**	− 2.73 (− 4.94; − 0.56)	**0.540**
ECBI intensity scale	− 22.8 (25.1) − 18.5 (− 88; 23) (− 29.3; − 16.4) *n* = 60	− 4.59 (19.28) − 5 (− 46; 44) (− 11.22; 2.07) *n* = 34	**0.0002**	− 18.2 (− 28.2; − 8.5)	**0.788**
ECBI Problem scale	− 4.83 (7.10) − 4 (− 26; 11) (− 6.67; − 3.00) *n* = 59	− 1.06 (3.99) − 1 (− 11; 7) (− 2.47; 0.37) *n* = 33	**0.0061**	− 3.77 (− 6.47; − 1.14)	**0.610**
Family burden of illness	− 3.03 (4.76) − 3 (− 20; 5) (− 4.25; − 1.81) *n* = 60	− 2.12 (3.91) − 2 (− 15; 7) (− 3.0; − 0.79) *n* = 34	0.35	− 0.916 (− 2.824; 0.952)	0.205
Relationship problems questionnaire	− 0.309 (2.873) 0 (− 7; 7) (− 1.080; 0.467) *n* = 55	− 0.517 (3.823) 0 (− 12; 11) (− 2.000; 0.923) *n* = 29	0.81	0.208 (− 1.278; 1.684)	0.064

Change from baseline to 10 weeks. PR-ESSENCE vs. Controls (FA set). No Imputation

For categorical variables *n* (%) is presented. For continuous variables Mean (SD)/Median (Min; Max)/(95% CI for Mean using the inversion of Fisher’s non-parametric permutation test)/*n* = is presented

For comparison between groups, the Mantel–Haenszel Chi Square test was used for ordered categorical variables and the Fisher’s Non Parametric Permutation Test for continuous variables. The confidence interval for the mean difference between groups is based on Fishers non-parametric permutation test. Effect Size is Difference in Mean/Pooled SD

*Investigator ratings* Change in CGI-S scores showed improvement for 63.3% (38/60) in the active group compared to 14.3% (5/35) in the control group, *p* < 0.0001.

*Child self-ratings* Treatment effects active vs controls measured by changes in BYI scores were largest on the disruptive behaviour subscale (ES 0.5, *p* = 0.044, mean difference − 9.18; 95% CI − 18.31, − 0.24), small on self-concept (ES 0.3, not significant, mean difference 7.04; 95% CI − 4.50, 18.82), and small to none on anxiety, depression and anger.

*Parent ratings* Treatment effects active vs controls seen in changes in SNAP-IV ratings were medium (ODD ES 0.54, *p* = 0.016, mean difference − 2.73; 95% CI − 4.94, − 0.56; ADHD ES 0.61, *p* = 0.0057, mean difference − 4.37; 95% CI − 7.40, − 1.33). On the ECBI scales effects were medium (Problem Scale ES 0.61, *p* = 0.006, mean difference − 3.77; 95% CI − 6.47, − 1.14) to large (Intensity Scale ES 0.79, *p* = 0.0002, mean difference − 18.2; 95% CI − 28.2, − 8.5). On the FBIM and RPQ scales effects were small to none.

Sensitivity analysis with stochastic imputation of missing data showed similar results on most measures, but somewhat stronger results on child BYI self-rating of disruptive behaviour (ES 0.63, *p* = 0.0026) and depression (ES 0.54, *p* = 0.012).

## Discussion

This RCT of the problem-resolution model PR-ESSENCE for children and adolescents with neurodevelopmental disorders/ESSENCE and challenging behaviour included assessments from various raters (blinded raters, children, parents, and therapists). Of the 136 subjects invited to participate, 108 were included in the trial, and 95 completed the randomized 10-week period (FA set). The main results in the study were that PR-ESSENCE treatment was superior to the control condition (Treatment As Usual) on the primary outcome measure CGI-I assessed by blinded raters, with a large effect size (ITT analysis ES 1.48, FA set ES 1.53). The treatment responder rate (subjects much or very much improved with only mild symptoms remaining, CGI-I score 1–2), was significantly higher in the active group (51.4%, 37/72) compared to controls (5.6%, 2/36). The FA set secondary outcome measures also showed treatment effects. On child self-ratings with the BYI the effect size was largest on the disruptive behaviour subscale (ES 0.5), smaller and non-significant (ES 0.3) on the self-concept subscale, and mall to none on anxiety, depression, and anger. On parent ratings the effect size was medium on SNAP-IV (ODD ES 0.54, ADHD ES 0.61), medium to large on the ECBI scales (Problem Scale ES 0.61, Intensity Scale ES 0.79), but on the FBIM and RPQ scales effects were small to none.

Compared with previous research on the CPS method, our trial shows equivalent to superior treatment effects on the primary outcome measure CGI-I. In US trials of CPS for children with ODD the proportion of subjects who were globally much or very much improved (CGI-I 1–2) were 70% (therapist ratings [7]) and 46.7% (assessment clinician ratings [8]). The blinded status of the CGI raters in our study strengthens the results. Since previous trials have shown that CPS results are at least equivalent to the well-established Barkley’s Parent Management Training method [8], this also supports the effectiveness of the PR-ESSENCE model.

An advantage of these problem-solving behavioural interventions, implemented with or without simultaneously given pharmacological treatment, is that they are all targeting the child’s cognitive deficits, within areas of flexibility and coping with emotional frustrations, and that they focus on developing and learning mutual problem-solving strategies. In our trial, also children with autism were included, i.e., children who, in addition to impairment in social interaction and communication, often have severe difficulties adapting to demanding situations. Combined cognitive, executive and theory of mind deficits make daily interaction especially problematic. An important aspect of the PR-ESSENCE intervention is that the children were carefully assessed and parents received information about their child’s specific cognitive difficulties and diagnoses. Knowledge about cognitive deficits underlying behavioural problems facilitates adults’ understanding and may significantly improve the child’s functioning.

It may be possible to implement the PR-ESSENCE model in Child and Adolescent Mental Health Services (CAMHS) under the supervision of therapists with expertise in neurodevelopmental disorders. General principles of the model have potential to be used in schools in everyday situations where problem resolution is needed. However, future research that examines the effectiveness of the model in community settings, as well as the amount of training and expertise needed to implement them as intended, is also recommended.

Several studies have reported impaired self-concept and self-esteem in children with ADHD and lower self-concept in older children with ADHD [23]. Early intervention helping the child to cope with frustrations and solve problems mutually would probably positively affect behaviour, functioning and self-esteem, as suggested by the results in our study.

To our knowledge, this is the first RCT of a problem-solving method including children with ADHD, autism and ADHD-autism combined. Other variants of behavioural interventions have been studied for many years. Cognitive Behaviour Therapy (CBT) models adapted for treating anxiety in children with autism [24–27] have showed promising results, although small samples and lack of controls make conclusions uncertain in some studies. Systematic reviews and meta-analyses of studies on CBT and parent or child training for children with ADHD report positive effects, but generally a low strength of evidence due to study limitations [28, 29].

### Strengths and limitations

Strengths of this study are the relatively large and well-defined clinical sample of children with complex neurodevelopmental disorders, the randomized design, the experienced therapists and assessors, the blinded ratings of the primary outcome measure, the assessment of outcomes from different sources (blinded raters, parents, therapists) and from the children’s own perspective, and the relatively low attrition during the treatment/control period (12%, 13/108 families discontinued during this period).

Limitations include the attrition of a larger number of participants in the PR-ESSENCE group (16.7%) than in the TAU group (2.8%) during the treatment/control period. Reasons varied (see p. 12), but some children expressed lack of motivation, and for some the therapy may have been too demanding. Further limitations were long-term attrition and missing data from some 45% of the families at the 6-month follow-up, and from 35% at the 1-year follow-up (the long-term results will be published elsewhere).

## Conclusions

This RCT demonstrated efficacy of the PR-ESSENCE problem-solving model for children and adolescents with complex neurodevelopmental disorders/ESSENCE such as ADHD and autism and severe challenging behaviour. Large effect sizes were found on improvements of global symptoms and functioning (CGI-I) and behaviour problems (ECBI), medium effect sizes on ADHD and ODD symptoms, and on children’s self-rating of disruptive behaviour. Treatment effects were at least equivalent to those in previous studies of well-established Parent Management Training programs and CPS.

## Data Availability

The data supporting the results from this study are available from the corresponding author (MJ) upon reasonable request.
